# Machine learning explains response variability of deep brain stimulation on Parkinson’s disease quality of life

**DOI:** 10.1038/s41746-024-01253-y

**Published:** 2024-10-02

**Authors:** Enrico Ferrea, Farzin Negahbani, Idil Cebi, Daniel Weiss, Alireza Gharabaghi

**Affiliations:** 1 Institute for Neuromodulation and Neurotechnology, University Hospital Tübingen (UKT), Faculty of Medicine, University Tübingen, 72076 Tübingen, Germany; 2 Center for Neurology, Department for Neurodegenerative Diseases, and Hertie Institute for Clinical Brain Research, University Tübingen, 72076 Tübingen, Germany; 3 Center for Bionic Intelligence Tübingen Stuttgart (BITS), 72076 Tübingen, Germany; 4 German Center for Mental Health (DZPG), 72076 Tübingen, Germany

**Keywords:** Quality of life, Predictive markers

## Abstract

Improving health-related quality of life (QoL) is crucial for managing Parkinson’s disease. However, QoL outcomes after deep brain stimulation (DBS) of the subthalamic nucleus (STN) vary considerably. Current approaches lack integration of demographic, patient-reported, neuroimaging, and neurophysiological data to understand this variability. This study used explainable machine learning to analyze multimodal factors affecting QoL changes, measured by the Parkinson’s Disease Questionnaire (PDQ-39) in 63 patients, and quantified each variable’s contribution. Results showed that preoperative PDQ-39 scores and upper beta band activity (>20 Hz) in the left STN were key predictors of QoL changes. Lower initial QoL burden predicted worsening, while improvement was associated with higher beta activity. Additionally, electrode positions along the superior-inferior axis, especially relative to the *z* = −7 coordinate in standard space, influenced outcomes, with improved and worsened QoL above and below this marker. This study emphasizes a tailored, data-informed approach to optimize DBS treatment and improve patient QoL.

## Introduction

The overarching goal of therapeutic interventions in chronic diseases is to improve the health-related quality of life (QoL). Because Parkinson’s disease (PD) has a substantial impact on multiple dimensions of QoL^1^ systematically monitoring QoL across the disease trajectory provides critical insights into those aspects that are particularly relevant to patients^2^. Such an approach can inform therapeutic strategies and patient-reported outcomes in ways that are not captured by clinician-rated assessments alone. However, the multifaceted construct of QoL, encompassing physical, mental, and social dimensions, introduces some complexity in identifying determinants that contribute to favorable outcomes during treatment. This complexity may pose a challenge in formulating optimal therapeutic recommendations for PD patients, which is well illustrated by the case of deep brain stimulation (DBS) of the subthalamic nucleus (STN).

STN DBS can provide long-term improvements in motor function for PD patients, but within a few years after surgery, QoL scores often decline to preoperative levels, which may reflect the progression of non-motor symptoms inherent to the neurodegenerative nature of PD^3^. In addition, there is also considerable variation in short-term QoL outcomes following STN DBS surgery, with up to half of the patients reporting no clinically meaningful improvement in their QoL following the procedure^4–7^. This puzzling situation has generated considerable interest in the search for preoperative predictors of postoperative QoL improvement^8^. Preoperative baseline QoL has been identified as the most consistent predictor of such an improvement with greater preoperative QoL burden being associated with greater postoperative QoL gains^5,9–11^. However, other studies have found the opposite relationship between pre- and postoperative scores^12,13^, thereby suggesting that other factors than the baseline patient status may influence postoperative QoL.

In a parallel line of research, factors related to the surgery itself and to patient-specific brain physiology have been investigated for their impact on postoperative outcome. Variations in the precise location of implanted electrode contacts within the STN have been shown to significantly affect outcome^14,15^. In addition, the magnitude of stimulation-induced neural responses within the STN was also critical for alleviating motor symptoms^16,17^, suggesting that comprehensive analysis of neuroimaging and neurophysiological data^18^ may be essential for understanding response variability after DBS. However, current research focusing on these variables to elucidate postoperative QoL variability is sparse^19^, and there is a lack of methodologies that integrate patient-reported, neuroimaging, and neurophysiological data to explain treatment response. However, a multimodal approach appears to be critical to elucidate the relationship between baseline and treatment-related factors that influence QoL outcomes, to facilitate patient stratification and counseling for surgery, and also to enable tailored interventions and refined surgical targeting.

The use of digital technologies and artificial intelligence is expected to unravel this complexity to some extent by providing insights into the many types of data available for monitoring disease progression and therapeutic interventions^20–22^. However, it is important to note that these innovative methods focus primarily on the classical motor symptoms of PD and often overlook QoL^23–26^.

In the present study, we aimed to address this knowledge gap by employing explainable machine learning methods to elucidate the response variability of STN DBS in terms of QoL. Preoperative patient-related data, intraoperative neurophysiological metrics, and perioperative neuroimaging findings were considered. We anticipate that our results will elucidate the relative impact of these various factors on outcomes, thereby informing the patient counseling and selection process as well as improving the surgical procedure and overall efficacy of the intervention.

## Results

### Framework overview

**Fig. 1:**
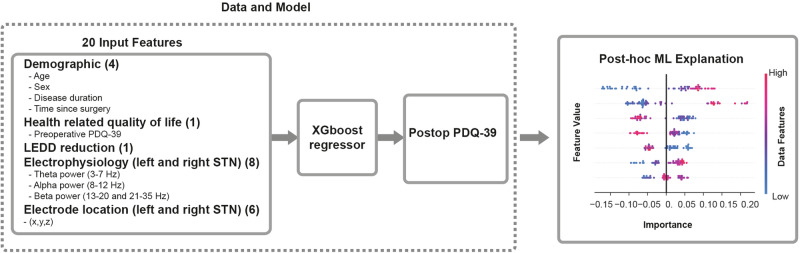
Framework overview. This diagram provides a comprehensive overview of our machine learning framework. On the left, we show the different types of data that are incorporated into the model. These include a range of inputs such as demographic information, clinical data, electrophysiological measurements, LEDD and electrode location. In the central part of the framework, the different data sets are integrated and processed via an XGBoost regressor model in a leave-one-out cross-validation fashion to predict post-operative PDQ-39 scores. Following the data processing and predictive modeling, we use SHAP (SHapley Additive exPlanations), an explainable machine learning tool, to explore the effective contribution of each feature to the model.

We used machine learning with the XGBoost Regressor and leave-one-out cross-validation to investigate complex factors influencing changes in QoL in PD patients, as indicated by the Parkinson’s Disease Questionnaire (PDQ-39). Our analysis included various data, including demographic information, preoperative PDQ-39 scores, electrode positions and intraoperative neural recordings along the implantation trajectory in standard space. We used this comprehensive data set to predict changes in PDQ-39 scores surgery after DBS. By applying SHAP (SHapley Additive exPlanations) values, we gained a post-hoc interpretive understanding of each factor’s contribution to the model’s predictions, enhancing our understanding of the variables influencing QoL improvement or deterioration (Fig. 1).

### Patient-related features

Sixty-three patients underwent deep brain stimulation (DBS) implantation surgery to alleviate symptoms associated with Parkinson’s disease. We compared the total scores on the Movement Disorder Society-Unified Parkinson’s Disease Rating Scale part 3 (MDS-UPDRS-3)^27^, which assesses the motor symptoms of Parkinson’s disease.

**Fig. 2:**
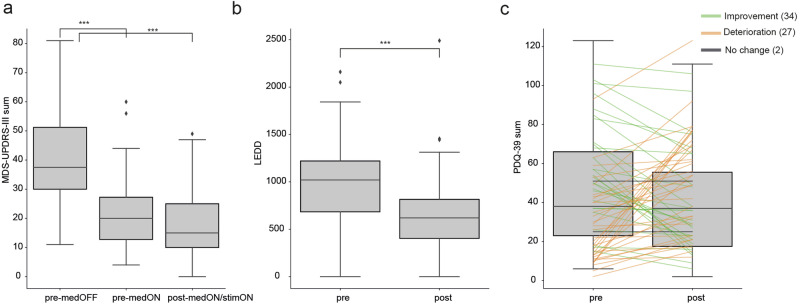
Deep brain stimulation effects. **a** UPDRS Part III (UPDRS3) total scores quantified before DBS implantation under off medication (pre-medOFF), on medication (pre-medON), and on medication with concurrent DBS stimulation (post-medON/simON). The ON conditions led to UPDRS3 improvements from the OFF condition by 49% (pre-medON) and 55% (post-medON/stimON), respectively (***p < 0.001). **b** Levodopa Equivalent Daily Dose (LEDD) before and after DBS implantation, showing a 32% reduction with DBS (***p < 0.001). **c** PDQ-39 scores assessed before and after DBS implantation, showing an average 4.67-point improvement with DBS (*p* = 0.183). The bars represent 95% confidence intervals.

Before surgery, levodopa (ON vs. OFF medication) improved motor symptoms with a significant (49%) reduction in MDS-UPDRS-3 (from 40.05 ± 14.70 to 21.35 ± 12.07 (mean ± std), T-statistic = 6.81, P_Bonferroni_adj = 2.73e-09). After surgery (20.6 months, STD: 15.23), this improvement persisted (55%), when levodopa medication was reduced and DBS was introduced (from 40.05 ± 14.70 – 18.04 ± 11.73, *T*-Statistic = 8.10, P_Bonferroni_adj = 5.64e-12, Fig. 2a). Consistent with previous literature^3^, DBS improved the MDS-UPDRS-3 score in the ON medication state by 3.31 points (post vs. pre-surgery, T-Statistic = 1.36, P_Bonferroni_adj = 0.52), while the dopaminergic medication was significantly reduced by 32% (levodopa equivalent daily dose (LEDD) from 983.48 ± 413.85 before to 666.33 ± 391.11 after surgery, *t* = 7.11, *P* = 1.37*E-9, Fig. 2b). Overall, these results demonstrate that DBS was effective in reducing the severity of motor symptoms with concomitant reduction in medication requirements.

In addition, we obtained PDQ-39 scores before surgery (in the dopaminergic medication ON state) and 20.6 ± 15.23 months (mean ± standard deviation) after DBS implantation (in the medication/stimulation ON state). The average PDQ-39 score improved by 4.67 from 45.75 ± 26.51 before to 41.08 ± 27.50 after DBS surgery (*t* = 1.35, *p* = 0.183, Fig. 2c). Thirty-four (54%) patients improved, two (3%) patients remained unchanged and twenty-seven (43%) patients worsened with regard to their PDQ-39 score. Twenty-six (41%) and twenty-two (35%) patients showed PDQ-39 changes of at least −4.72 and +4.22 points indicating minimal clinically important improvement and worsening, respectively^28^.

For further analysis, treatment-related changes in the PDQ-39 were quantified by subtracting the postoperative from the preoperative score and dividing this difference by the sum of both scores. This normalization process constrained the scores to a range between −1 and 1. Scores closer to 1 indicate improvement, while those approaching −1 indicate deterioration in QoL. We also included additional patient characteristics known to influence QoL, i.e., age (65.83 ± 8.23 years), disease duration from diagnosis to postoperative assessment (11.38 ± 4.79 years), sex (47 males and 16 females), time since surgery (20.6 ± 15.23), LEDD ratio (0.70 ± 0.31), preoperative PDQ-39 score (45.75 ± 26.51), and normalized PDQ-39 change (0.08 ± 0.35).

### Electrophysiological features and electrode locations

**Fig. 3:**
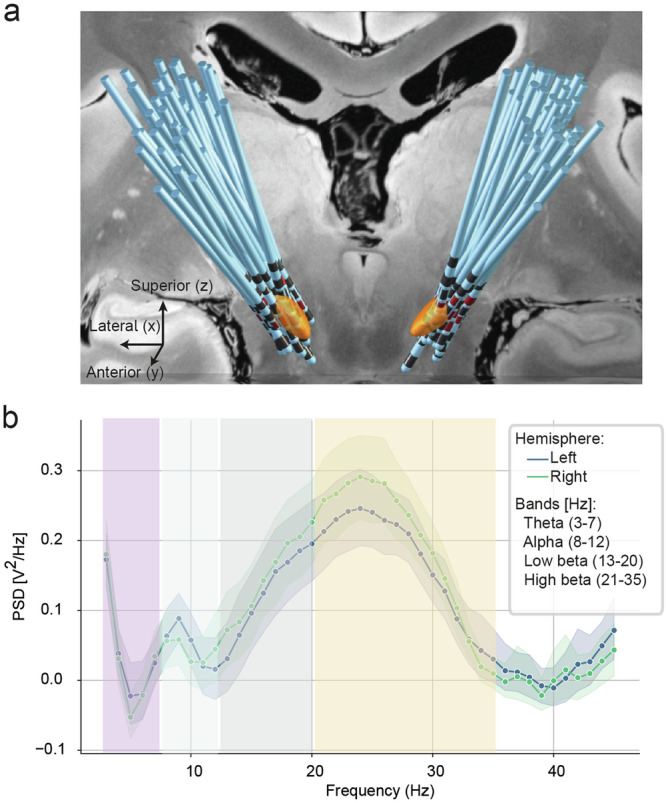
Electrophysiological features and electrode positions. **a** Reconstructed electrode leads in the subthalamic target region in the Synthesized FLASH25 100 Micron MRI in MNI Space^63^. **b** PSDs with the aperiodic 1/f component removed were averaged for the left (blue) and right (green) hemispheres across all patients. Additionally, theta, alpha, low beta (13–20 Hz), and upper beta (21–35 Hz) frequencies are shaded in pink, light blue, gray, and orange, respectively. Solid lines represent mean values, whereas shaded areas represent 95% confidence intervals.

During each implantation trajectory, we collected continuous electrophysiological data using a novel online mapping technique over the DBS electrode lead^29^. For each patient, 9.94 ± 3.87 (mean ± std) recording depths were collected, cumulating to a total of 1190 recording sites across the entire patient cohort. We then reconstructed electrode positions by co-registering preoperative MRI with postoperative CT images to determine the locations of electrophysiological recordings (Fig. 3a). Each electrode contact recording was then re-referenced to the uppermost contact. For our model, we used periodic activity in different frequency bands: theta (3–7 Hz), alpha (8–12 Hz), lower beta (13–20 Hz) and upper beta (21–35 Hz). We refined the power spectral density at each recording site by removing 1/f components (dashed lines), which facilitated the isolation of distinct oscillatory activity peaks (solid lines) (Fig. 3b). Each recording was then annotated with the corresponding anatomical structure in standard space^30^, allowing us to determine which recordings along the trajectory were within the STN. The electrode locations of the therapeutically active contacts were determined as the x/y/z coordinates in standard MNI space.

For model prediction, we used different electrophysiological recordings to consider different scenarios, where physiological information could improve the therapeutic procedure: along the implantation trajectory to improve electrode placement and intraoperative targeting^29^; averaged over all contacts at the final electrode lead position to inform adaptive DBS, where LFP at neighboring electrode contacts is used to control the active contact stimulation^31^; and at the therapeutically active electrode contacts to accelerate contact selection for continuous treatment^29^.

### Model performance

**Fig. 4:**
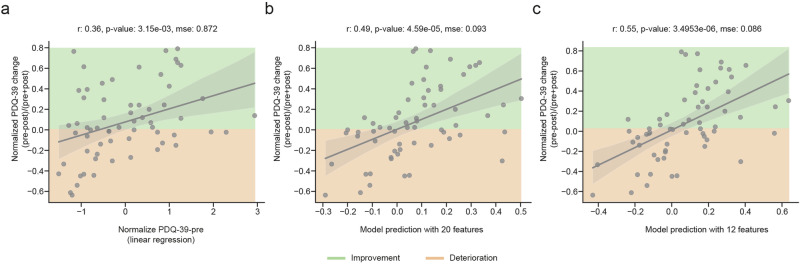
Model performance. **a** This figure illustrates the correlation between preoperative PDQ-39 scores and postoperative improvement. The preoperative scores were standardized using Z-scores, while the variational scores were calculated as normalized differences. This was done by subtracting the postoperative scores from the preoperative scores and then dividing the difference by their sum. **b** Prediction of pre- postoperative improvement from the model based on multimodal information from all 20 baseline features in a leave-one-out cross-validation fashion. **c** Same as the middle panel, but with a reduced number of features (12) after preliminary SHAP analysis. The shaded area around the regression line represents the 95% confidence interval.

The analysis demonstrated a statistically significant correlation between preoperative PDQ-39 scores and normalized PDQ-39 changes (Fig. 4a, *p* = 3.15e-3, *r* = 0.36, mse = 0.872). This indicates that patients with greater preoperative burdens experienced more substantial postoperative improvements. The incorporation of additional factors, including demographics, electrophysiological data of each hemisphere, time since surgery, LEDD, and electrode contact position, into the models yielded further insights.

The electrophysiological data was initially evaluated separately for each hemisphere in the three scenarios described above: along the implantation trajectory (Fig. 4b, *p* = 4.59e-5, *r* = 0.49, mse = 0.093), averaged over all contacts at the final electrode lead position (*p* = 4.28e-3, *r* = 0.36, mse = 0.109) and at the therapeutically active electrode contact (*p* = 4.39e-3, *r* = 0.35, mse = 0.11).

The same analysis was then conducted on the average of both hemispheres along the implantation trajectory, resulting in similar findings, but to a lesser magnitude (*p* = 1.28e-3, *r* = 0.40, mse = 0.106).

Additionally, a model considering average electrophysiological features by grouping them according to the presumed most and least affected hemispheres, was also investigated (*p* = 6.54e-3, *r* = 0.34, mse = 0.11).

These findings indicate that, regardless of the chosen approach, physiological information can contribute to model prediction with a high degree of reliability, and that hemispheric differences play an important role in this process. The optimal physiological approach (Fig. 4b, *p* = 4.59e-5, *r* = 0.49, mse = 0.093) was then subjected to further investigation by additional modifications. When predicting the absolute instead of normalized changes between preoperative and postoperative PDQ-39 scores, the findings remained stable (*p* = 2.97e-4, *r* = 0.44 mse = 0.82).

When using the distance from the optimal target site (as defined by Tödt et al.^19^) and stimulation amplitudes instead of electrode coordinates the prediction also remained significant (*p* = 2.61e-4, *r* = 0.44, mse = 0.10).

Despite these variations in the models, all demonstrated significant decoding performance, retaining significance after adjusting for multiple comparisons (corrected alpha = 7.14e-3). This consistency across models underscores the robustness of the main feature importance identified, as detailed in Supplementary Table 1. The analysis continued with the model showing the highest Pearson correlation coefficient, i.e., *r* = 0.49.

### Model explanation

We used Shapley values to assess the importance of each model feature^32^. These values illustrate the role of each feature in influencing the model’s output by modifying the input, thus providing insights into the model’s predictive dynamics. In our analysis, we computed Shapley values for all model features and compared their mean absolute values. To increase model predictability, we reduced our initial model by removing eight of the least important features (i.e., the eight lowest Shapley values) in the model. This further improved the model performance from *r* = 0.49 to 0.55 (Fig. 4c).

**Fig. 5:**
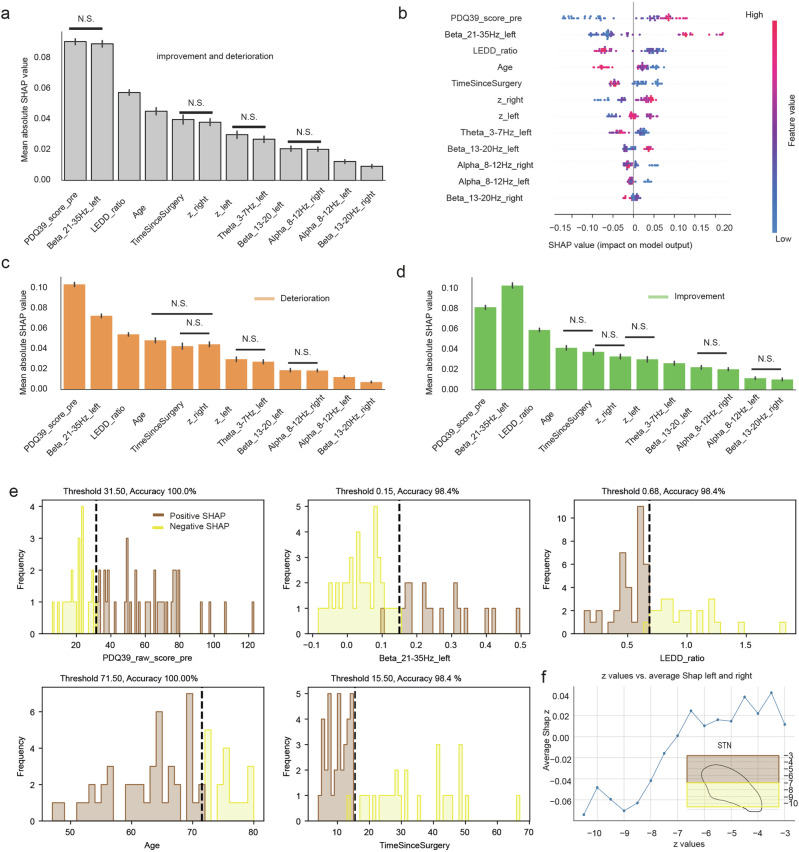
Shapley values. **a** Histograms of mean absolute Shapley values: The histograms show the mean absolute Shapley value for each feature, accompanied by cross-validated confidence intervals. An ANOVA performed on the twelve main features shows a significant effect of each feature on the value. In addition, a multiple comparison analysis among these features also indicates significant differences among them. In the graph, the features are arranged in descending order of their importance. Unless indicated with N.S., all multiple comparisons were significant at an alpha level of 0.005. **b** Beeswarm plot: It provides a detailed visualization of the distribution of Shapley values for each feature in our model. In this plot, each dot represents the Shapley value of a feature for an individual prediction, allowing us to observe the spread and density of these values across different instances. The features are listed on the *y*-axis, and their corresponding Shapley values are plotted along the x-axis. The positioning of the dots along the x-axis indicates the magnitude and direction of each feature’s contribution: dots located to the right of the center line represent a positive contribution to the prediction, while those to the left indicate a negative contribution. A key advantage of a Beeswarm plot is its ability to display the distribution of values without overlap, making it easier to identify patterns, outliers, and the overall impact of each feature. **c** Same as in (**a**), but separated for patients that worsened and (**d**). improved. The two graphs illustrate the difference in the contribution of the main twelve features. **e** Each panel illustrates the distributions of the features, color-coded by their positive (brown) or negative (yellow) contribution to the SHAP value. The threshold value displayed at the top represents the value that most effectively separates the two groups with the indicated accuracy using an SVM decoder. **f** The analysis of electrode location along the z-axis (depth), when considered in relation to SHAP scores associated with improvement and deterioration averaged over the two stimulating electrodes, indicates that electrode placements above −7 in standard MNI space were beneficial for postoperative improvements. Conversely, locations below this threshold were found to be detrimental. The inset of this panel depicts the two demarcated areas in relation to the STN.

Notably the preoperative PDQ-39 score along with the upper beta band activity emerged as the two most prominent features (Fig. 5a). In addition, postoperative levodopa reduction (LEDD ratio), age, time since surgery together with the depth position of the active electrode contacts emerged as features followed by the other electrophysiological features.

To further explore how each feature influenced the predictions, we conducted an analysis of variance (ANOVA) to assess the effect on the mean Shapley values. The ANOVA showed a statistically significant effect, suggesting distinct contributions from each feature (11 degrees of freedom, *F*-value of 720.99, *p*-value < 0.001).

A multiple comparison analysis was then performed using Tukey’s Honestly Significant Difference (HSD) test to distinguish differences in Shapley values between feature groups, with a Family-Wise Error Rate (FWER) set at 0.05. Non-significant (N.S) differences are reported in Fig. 5a, while all other multiple comparisons show significant differences with adjusted *p*-values < 0.001 (see also Supplementary Table 1). This shows that upper beta activity of the left STN and preoperative PDQ-39 scores are significantly different from all other features, highlighting their substantial impact on postoperative PDQ-39 predictions. In addition to the two main features, the LEDD ratio, age, and time since surgery had a high influence on the final prediction. Among the electrode position features, only active contact depth (z coordinate) influenced the final score. Shapley values not only quantify the contributions of each predictor (Fig. 5a) but also clarify their directional relationship with the outcome (Fig. 5b). For example, an increase in the upper beta band within the left STN positively correlated with PDQ-39 improvements. In addition, higher preoperative PDQ-39 scores, i.e., greater burden, generally led to greater postoperative improvements (see also Fig. 5b), emphasizing the value of preoperative patient-reported information. The LEDD ratio influenced the final score such that a greater reduction in medication (indicative of effective DBS) corresponded to more significant improvements. Age and time since surgery also influenced the scores inversely, i.e., the younger the age or the shorter the time since surgery, the greater the improvements observed.

To further investigate how different features affected positive and negative scores, an additional analysis was performed distinguishing between QoL deterioration (Fig. 5c) and QoL improvement (Fig. 5d). The results indicate that the two main features contributed differently to the improvement and deterioration scores. Specifically, baseline PDQ-39 was more relevant in predicting deterioration, whereas upper beta activity in the left STN was more relevant in predicting improvement. In fact, a multiple comparison between these two features showed a significant difference for both positive and negative contributions (*p* < 0.001).

In order to obtain actionable insights, the SHAP values were correlated with the feature values. This was followed by the application of a support vector machine (see methods) in order to determine thresholds for each of them that determined a positive contribution to the model prediction (Fig. 5e). This analysis yielded cutoff values for each feature independently with high accuracy. A larger QoL burden before surgery, as indicated by a PDDQ-39 baseline score of >31.5 points, and a power spectral density in the upper beta band (21–35 Hz) of the left STN of >0.15 V^2^/Hz were identified as significant contributors to the prediction of improvement following DBS. Furthermore, a reduction in LEDD of >32% (i.e., LEDD ratio < 0.68) and an age at surgery of <71.5 years were identified as additional relevant contributors to the model indicative of improvement.

Analysis of active contact locations (mean ± std, x left: −12.53 ± 1.24, x right: 11.59 ± 1.34; y left: −12.76 ± 1.58, y right: −12.34 ± 1.49, z left: −6.57 ± 1.40, z right: −6.49 ± 1.70) in relation to PDQ-39 changes, indicated that optimal electrode placement contributed to therapeutic success (Fig. 5f). Specifically, postoperative improvements in QoL were associated with active contact positions along the dorso-ventral (superior-inferior) axis of the STN. When considering the contribution of both electrode leads, active contact positions above and below *z* = −7 in standard MNI space positively and negatively influenced QoL, suggesting the importance of precise targeting within the upper part of the STN for improved therapeutic efficacy (Fig. 5f). These findings confirm that specific physiological and electrode position characteristics significantly influence patient outcomes and underscore the need for personalized approaches to therapeutic planning and delivery.

### Feature ablation analysis

To further validate our findings and to rule out the possibility that correlations between features affected the reliability of our feature importance method, we performed an additional ablation analysis on the reduced model. In this analysis, we selectively removed features and observed the effect on model performance, specifically in terms of Pearson’s coefficient. Our results showed that removing the preoperative PDQ-39 score decreased the model performance from *r* = 0.55 to *r* = 0.36. Similarly, removing upper beta activity in the left STN decreased performance to *r* = 0.37. These results indicate that these two features are essential for optimal model performance. When LEDD reduction, an indicator of DBS efficacy in alleviating motor symptoms, was removed from the model, there was no relevant decrease in model performance (*r* = 0.52). This suggests that the contribution of upper beta activity to the model was independent of DBS efficacy in alleviating motor symptoms, as reducing LEDD did not affect the model as much as removing the upper beta activity.

An additional analysis was conducted to control for the possibility that upper beta power contributed to the model as a surrogate of motor symptom severity only. To this end, we ran a model in which we substituted the upper beta activity feature with the baseline UPDRS-3 score. If both features were to represent the same clinical domain in the model, the model performance should remain consistent. However, when the model was trained with the UPDRS-3 score instead of the upper beta activity, this resulted in a notable decline in performance (*r* = 0.37). In this model, the UPDRS-3 score ranked second to last in terms of importance, while the beta power was identified as the top feature in the initial model. These observations collectively suggest that the upper beta activity in the left STN is a significant predictor of postoperative QoL change, independent of the baseline motor severity.

Next, we performed a removal of age (*r* = 0.50), time since surgery (*r* = 0.53), and averaged electrode depths (*r* = 0.52). Removing electrode depths did not decrease the model performance as much as removing the upper beta activity, and also indicated a significant independence between the beta activity and electrode position components. Finally, removal of the remaining electrophysiological features did not reduce performance, suggesting a functional specificity of the upper beta activity.

Taken together, these results support and parallel the analysis of Shapley values, indicating different contributions of these features to the model and effectively excluding significant confounding correlations among them.

## Discussion

In this research, we sought to address the considerable variability in QoL outcomes following DBS for PD, a critical issue given the primary goal of improving health-related QoL in chronic disease. Utilizing explainable machine learning via the XGBoost Regressor and SHAP analyses, we analyzed a wide range of variables, including demographic details, preoperative QoL assessments, electrode positions, LEDD reduction, time since surgery, and intraoperative neural recordings. Our study highlights the significant role of both baseline patient-reported scores and the precise positioning of electrodes within the STN in predicting QoL outcomes after surgery. Notably, the individual neurophysiological characteristics showed the highest predictive capability for QoL improvement, surpassing other examined factors. These findings underscore the complex interplay between patient-reported data, accurate electrode placement, and individual neurophysiological markers as critical determinants in the success of DBS procedures.

Initially, DBS for PD primarily focused on motor symptoms, evaluating therapy effectiveness predominantly through clinical metrics like the motor examination part III of the Unified Parkinson’s Disease Rating Scale^27^. However, a shift towards a more holistic view has emerged, recognizing the importance of non-motor aspects of the disease. The use of patient-reported outcome measures such as the PDQ-39 has become increasingly relevant, providing insights into clinically significant changes post-therapy^28^.This broader approach can offer valuable information for therapeutic strategies beyond what is obtained from clinician-based evaluations alone. Identifying predictors of QoL changes is crucial, especially since a significant portion of patients do not report meaningful QoL improvements in the course of treatment^4–7^.

Consistent with prior research, our study confirms the strong predictive value of preoperative QoL on postoperative changes, surpassing other baseline patient information like age, sex, or disease duration. We observed that patients with greater preoperative QoL burdens tend to experience more substantial QoL gains post-intervention, similar to previous studies^5,7,9–11,30^. Notably, lower preoperative QoL burden was more predictive of QoL deterioration at an average of 20 months after surgery than any of the other features examined (Fig. 5c), providing valuable information for patient counseling. Moreover, the proposed method also enabled the identification of threshold values to discriminate between the positive and negative contributions of specific features to the postoperative outcome (see Fig. 5e). Specifically, a PDDQ-39 score exceeding 31.5 points was identified as a significant predictor of improvement. Furthermore, the model’s prediction of improvement was influenced by the age at surgery being below 71.5 years, although this effect was considerably less pronounced. These findings complement previous work on predicting QoL after DBS:

In the secondary analysis of the EARLYSTIM study, a randomized clinical trial comparing STN DBS with best medical treatment over a 24 month follow-up, researchers examined baseline factors like age, disease duration, PDQ-39 scores, duration of motor complications, and disease severity (assessed by UPDRS-III and UPDRS-IV scores) to identify predictors of PDQ-39 change. Remarkably, only the baseline PDQ-39 scores, not the other baseline characteristics, correlated with PDQ-39 changes in both treatment groups^11^.

Additionally, an observational study reported that patients with more severe preoperative QoL impairments and a heavier non-motor symptom burden exhibited more significant postoperative QoL improvements and a higher likelihood of experiencing clinically relevant changes 6 months after DBS surgery. Notably, stratification based on motor progression or levodopa response did not predict these outcomes^7^.

An increasing number of studies are highlighting the positive non-motor effects of STN DBS in mental, behavioral and autonomic domains^33–36^, indicating an overall postoperative reduction in non-motor symptom burden from baseline, which aligns with improved QoL^7,37^.

Given that non-motor aspects of PD often predict postoperative QoL outcomes more effectively than motor aspects, future studies should consider including more detailed assessments of non-motor symptoms to refine patient selection and enhance postoperative QoL outcomes^6,38^.

Analysis of the electrode lead placement in relation to PDQ-39 changes showed that electrode contact positions along the dorso-ventral axis of the STN were predictive of outcome. Specifically, active contact positions above and below *z* = −7 in standard MNI space had positive and negative effects on QoL, highlighting the importance of accurate targeting within the STN for improved treatment efficacy. Should further studies confirm these findings, it would open up promising avenues beyond the selection of the right patients for DBS therapy. It could lead to treatment-related modifications of surgical targeting to optimize QoL outcomes.

However, existing studies investigating electrode placements to clarify variability in postoperative non-motor symptoms and/or QoL remain contradictory^10,15,19^:

Initial findings indicated that more anterior, medial, and ventral electrode locations were related to more beneficial non-motor outcomes and QoL improvements^10^. Subsequent research by the same team corroborated this pattern in a broader cohort, linking improvements in mood/apathy, attention/memory, and sleep directly to QoL outcomes after subthalamic DBS^15^. Notably, these postoperative improvements exhibited a clear dependency on the electrode placement, with positions relevantly more ventral, or nearer the lower STN border, than those commonly reported for motor improvements^14,39–41^.

A contrasting perspective emerges from a recent investigation of the EARLYSTIM cohort^19^, which identified the upper center of the STN as the prime site for motor improvement, aligning with previously identified motor sweetspots^14,39–41^. However, the study suggested that the optimal region for enhancing QoL is posterior and superior to this optimal motor spot, i.e., near the upper STN border.

Our study contributes a new viewpoint, associating opposing effects on QoL with a specific depth (*z* = −7) in standard MNI space. Given the varied findings from available neuroimaging studies, a conclusive sweet spot for QoL improvement remains elusive. However, the neurophysiological insights from our research add a complementary dimension to this ongoing exploration, highlighting the potential of physiologically informed refinements of electrode placement.

Basal ganglia beta activity (13–35 Hz) in PD stands out as one of the most well-documented pathological brain rhythms^18^. Considered a biomarker for the brady-/hypokinetic motor state, exaggerated beta activity in the basal ganglia correlates with the severity of motor symptoms such as bradykinesia and rigidity^16,17,42,43^. Moreover, intraoperatively identified beta oscillations are correlated with motor outcomes^44^. Their potential role for non-motor symptoms of PD is less well studied.

Recent research^45^ has indicated a correlation between intraoperative neurophysiological recordings in the STN with both motor and non-motor outcomes. Analysis of microelectrode recordings of the STN spiking activity pinpointed criteria indicative of beneficial outcomes: notably high normalized neuronal activity, significant STN width, and a high relative proportion of the STN dorsolateral oscillatory region.

Our current research complements these findings with an alternative intraoperative technique using LFP recordings every millimeter along the surgical trajectory directly from the macrocontacts of the DBS leads to guide lead placement. Previous work has shown that this method can inform and intraoperatively adjust surgical lead placement, and also improve stimulation programming procedures^29^. The threshold for the power spectral density (above 0.15 V²/Hz) in the upper beta band (21–35 Hz), which was identified as a significant predictor of improvement following DBS in the present study, may inform even further refined procedures in the future. This physiologically informed approach can now be extended through sensing-enabled devices that allow chronic recordings well beyond implantation^46^. Using these novel devices, recent work has shown that dopaminergic medications primarily affect lower beta frequencies, whereas DBS provides a greater suppression of upper beta frequencies^47^, a finding that may be extended by the results of the present study.

We demonstrate that the upper beta band may serve as a biomarker for QoL improvement beyond its recognized role in motor symptoms. The fact that this biomarker was significantly more predictive of QoL improvements than demographics, electrode positions and initial QoL scores highlights its clinical importance and warrants further investigation of its relationship with non-motor symptoms.

We also included postoperative LEDD reduction (with concurrent MDS-UPDRS-3 improvement) in the analysis as an indicator of DBS efficacy in alleviating motor symptoms. Interestingly, LEDD reduction contributed to the prediction of QoL and a reduction in LEDD of >32% was identified as a significant contributor to the model’s predication of improvement. However, this predictor was significantly outperformed by upper beta band activity and baseline PDQ-39 scores. Furthermore, when LEDD reduction was removed from the model, there was no significant decrease in predictive performance, in contrast to the effect that the removal of upper beta band activity and baseline PDQ-39 had on model performance. Similarly, when upper beta band activity was replaced by MDS-UPDRS-3, the model performance dropped relevantly. These findings suggest that intraoperative upper beta activity may be more than a physiological surrogate of motor severity only, and may contribute to the prediction of QoL outcomes independent of the motor improvements achieved.

In addition, we found that upper beta activity in the left STN was particularly predictive of QoL improvement. This finding is consistent with previous observations of hemispheric asymmetry of neurodegeneration and its influence on the non-motor outcome of STN DBS^48,49^. Specifically, a previous study showed that DBS had a beneficial effect on quality of life primarily in PD patients with predominantly right-sided motor symptoms and presumed left cerebral pathology(Voruz et al.^48^). In addition, an electrophysiological study found that oscillatory beta-band activity in response to emotional stimuli was predominantly in the left STN, independent of the lateralization of motor symptoms^50^. In summary, our analysis extends previous work by suggesting a biomarker for QoL improvements following DBS while further emphasizing the importance of distinguishing hemispheric differences to elucidate non-motor symptoms.

The limited size of our sample precluded the use of more sophisticated models and clustering techniques. Future studies should involve larger cohorts and include additional variables to better distinguish the direct and indirect effects of DBS on QoL. Therefore, while our findings provide valuable insights, they are preliminary.

Nevertheless, our methodology is both accessible and replicable, and lays the groundwork for future research. As directional DBS technology, which allows for targeted stimulation of specific STN subregions, is now widely available, our approach may become increasingly relevant. In addition, emerging sensing technologies promise to facilitate the monitoring and targeting of specific frequency bands, potentially optimizing stimulation programming. Continued exploration of the clinical relevance of targeting specific biomarkers and STN subregions is essential. This should be coupled with careful monitoring of clinical parameters and potential non-motor effects to refine and improve DBS treatment strategies.

This study highlights the significant variability in quality-of-life outcomes following deep brain stimulation for Parkinson’s disease and underscores the need for personalized treatment approaches. Utilizing explainable machine learning techniques, the study analyzed a variety of factors including demographic, patient-reported, neuroimaging, and neurophysiological data to understand this response variability. Results indicate that both preoperative QoL and upper beta band (>20 Hz) activity in the left STN significantly predicted QoL changes following DBS. Worsening and improvement were particularly predicted by lower initial QoL burden and higher beta activity, respectively. In addition, electrode lead placement along the superior-inferior axis also influenced outcome, with improved and worsened QoL related to active contact positions above and below the *z* = −7 coordinate in standard space. The study highlights the importance of a multifaceted data integration strategy and advocates for a detailed, data-informed approach to optimizing treatment and improving the QoL for PD patients.

## Methods

### Patients

This study was conducted in accordance with the Declaration of Helsinki and included sixty-three consecutive Parkinson’s disease patients (one hundred twenty-six electrode leads in total), all of whom received bilateral segmented DBS electrode leads (6170, Abbott Laboratories, Lake Bluff, IL). Our primary outcome measure, the PDQ-39, a widely used patient-reported scale in Parkinson’s disease, was acquired during the patients’ hospital visits both before DBS implantation surgery and at follow-up. The PDQ-39 covers eight domains: mobility, activities of daily living, emotional well-being, stigma, social support, cognition, communication and bodily discomfort^51^. The dorsal-most and ventral-most levels of the electrode leads had omnidirectional contacts, while the two intermediate levels contained three segments each (frontal, medial, lateral). All participants underwent DBS implantation after overnight withdrawal of dopaminergic medication, and no surgical complications were reported. Ethical approval (781/2015B02) for this research was granted by the University Hospital Tübingen Ethics Committee. The need for informed consent was waived by the ethics committee since all data used in this study was acquired in the context of standard clinical care.

### Implantation procedure

For each patient, we determined the location of the STN using neuroradiologic magnetic resonance imaging. Leads were implanted strategically to position the two segmented electrode levels, i.e., the second and third highest levels, within the STN, resulting in 12.7% clinically active contacts at the dorsal-most, 75.4% at the second-highest level, 11.1% third-highest and 0.8% at the ventral-most level. We based the implantation depth on electrophysiological mapping, in line with methods outlined previously^29^. Only one contact level was activated in the clinical setting.

### Electrophysiological recordings and analysis

Neural recordings were acquired from the subthalamic nucleus (STN) region during deep brain stimulation (DBS) surgeries as previously described^29,52^. Specifically, monopolar local field potentials (LFPs) were recorded from the DBS electrode lead as it was incrementally advanced along the implantation trajectory. Across all 126 trajectories, we performed simultaneous LFP recordings from 8 monopolar macroelectrode recordings, capturing both omnidirectional and segmented lead contacts at every millimeter of the surgical path. Each level was recorded for 30 s at a sampling rate of 5500 Hz (NeuroOmega, Alpha Omega Engineering, Israel). Recording began with the ventral-most contact situated 8–10 mm above the tentative target, and each subsequent dorsal contact level was simultaneously recorded at +2 mm, +4 mm, and +6 mm above. Simultaneously, the power spectral density (PSD) was computed and displayed online (as shown in Fig. 1). In addition, data were transferred via ethernet to an additional computer for real-time electrophysiological monitoring using a custom Python (version 3.10) script.

Offline, LFP signals were extensive processed using a custom Python (version 3.9.7) script and the MNE package^53^. Signals were first resampled to 2200 Hz and then filtered with a 4th order Butterworth high pass filter at 1 Hz. Channels with anomalous signal deviations were identified and excluded. All channels were then re-referenced to the dorsal-most contact, with line noise notched out. The signals were then low-pass filtered at 450 Hz and segmented into 3 s epochs with a 2 s overlap. Epochs containing artifacts were identified and removed using the Riemannian potato method^54^.

Power spectral densities (PSDs) for each epoch were calculated using the Welch method and then averaged. To discriminate between aperiodic and periodic activity, the FOOOF package^55^ was used, focusing on the periodic component to calculate PSDs across the theta (3–7 Hz), alpha (8–12 Hz), lower beta (13–20 Hz), and upper beta (21–35 Hz) bands (Fig. 3b). The FOOOF algorithm was applied to each electrode contact independently before averaging the findings. At each depth, for segmented contacts, power band features and corresponding locations were averaged to produce a composite feature per level. This comprehensive approach allowed for a nuanced analysis of the electrophysiological data along the implantation trajectory.

The step-wise LFP recordings were used during implantation for electrophysiological mapping to inform lead placement as described previously^29^. For prediction, only the average LFP activity for each frequency band was used and, depending on the model, included the average of all recordings along the implantation trajectory within the STN (original), the average of the recordings at the final lead position of all contacts (final position), or the therapeutically active contact only during PDQ39 assessment (stimulation site). All of these models considered the left and right STN recordings separately. To further investigate the reliability of model prediction, the same analysis was also performed on the average of both hemispheres along the implantation trajectory (average of hemispheres), after grouping them according to the presumed most and least affected hemispheres (affected hemisphere), and when predicting the absolute rather than normalized changes between preoperative and postoperative PDQ-39 scores (absolute score).

### Imaging

Co-registration was performed between preoperative MR images acquired on a 1.5 T scanner (Siemens MAGNETOM Aera) and postoperative CT images (Siemens SOMATOM Definition AS+) with spatial resolutions of 0.5 × 0.5 × 0.5 mm for 23 patients, and 0.5 × 0.5 × 3 mm for 38 patients. All acquired images were uniformly resampled to an isotropic resolution of 1 mm in all planes using a B-spline interpolation method.

For electrode localization, we used the Lead-DBS pipeline v2.6^56^ with co-registration of pre- and postoperative images facilitated by Advanced Normalization Tools (ANTs)^57^ and Statistical Parametric Mapping (SPM)^58^ software, which also co-registered T1 to T2 and FLAIR sequences to confirm electrode placement in native anatomical spaces. Preoperative T1 images were then normalized to the MNI ICBM 2009b nonlinear stereotaxic space using ANTs, the coarse mask protocol^59^ was used to correct for brain shift, and electrode locations were manually refined to account for visible artifacts.

We assigned power-band features to the identified electrode locations from the Lead-DBS pipeline, adjusting for implanted depth and all recording depths along the trajectory. Each recording location was further categorized according to the DISTAL atlas^30^, distinguishing between inside and outside the STN using a custom Python script and the PyVista package^60^. As a result, we created a dataset that included electrophysiological data, 3-dimensional coordinates in MNI space, and atlas-based anatomical annotations, providing a comprehensive framework for subsequent analysis.

### XGBoost Regression Model

We formulated a regression problem to predict the improvement/deterioration of PDQ-39 from various features using an XGBoost regression model. All input features were standardized using *z*-score normalization before training the model.

We used a nested leave-one-out optimization method, similar to that described previously^61^, to best tune the model hyperparameters. Briefly, each model in the leave-one-out process was optimized in a k-fold cross-validation fashion (*k* = 6). We used the Hyperopt library^62^ with the Bayesian optimization search algorithm to sample the hyperparameter space. Parameters tuning was conducted in a sequential order, starting with the number of estimators, then min_child_weight along with max_depth, gamma, followed by subsample, and colsample_bytree. Each parameter was sampled 300 times, and the parameter with the best mean squared error in the validation set was retained in the final model. We identified the following ranges for the parameters used in the optimization (see Supplementary Table 2).

### Shapley details

We used the SHAP (SHapley Additive exPlanations) library in Python (https://shap.readthedocs.io/en/latest/), integrated with an XGBoost regressor model, to elucidate the significance and impact of each feature in our model. By calculating the mean of the absolute values from the shap_values.values for each feature, we quantified their importance. In addition, we utilized SHAP’s Beeswarm and Decision Plot functions, which were instrumental in graphically representing the influence of SHAP values on individual features across all observations, thereby illustrating the specific ways in which each feature affects the model’s predictions.

### Statistics

We used the leave-one-out cross-validation technique to assess the performance of our model, with accuracy measured by the Pearson correlation coefficient between predicted and actual postoperative PDQ-39 variational scores. The significance of the model’s fit was assessed by calculating the *p*-value, with a threshold of 0.05 set for statistical significance. These procedures were performed using the stats module of the Scipy library.

For a detailed statistical examination of the differences between the Shapley values of different features, we used leave-one-out cross-validation to derive cross-validated distributions of the mean Shapley value for each feature. The top three features, based on performance, were then subjected to a comparative analysis. An initial analysis of variance (ANOVA) tested the effect of different features on the mean Shapley value, using the ‘ols’ function from the ‘statsmodels.formula.api’ module. The resulting *F*-values and *p*-values were calculated using the ‘anova_lm’ method from ‘statsmodels.api’. To further analyze the differences between the features, multiple comparisons were performed using Tukey’s Honestly Significant Difference (HSD) test via the ‘pairwise_tukeyhsd’ function in the ‘statsmodels.stats.multicomp’ module. This approach allowed for a controlled assessment of significant differences between the means of different groups, taking into account the family-wise error rate.

### Ablation analysis

In the ablation analysis, specific features were removed in order to assess their impact on the model’s performance. After excluding each feature, the model was retrained and its performance was re-evaluated to determine the importance of each feature to the overall accuracy and effectiveness of the model. In addition, in one control analysis, the upper beta activity of the left STN was replaced by the MDS-UPDRS-3, and the model was reevaluated to determine whether the former could be considered a physiological surrogate of the latter.

### Support vector machine

To find feature values that best separate positive and negative contributions to the final score, we used a support vector machine (SVM) decoder with the scikit-learn library in Python. Since the data are one-dimensional, we used a linear kernel and a high regularization parameter (C = 1000). These choices allowed us to separate positive and negative scores with high accuracy.

## Supplementary information

Supplementary Information

## Data Availability

Data are available upon request from the first author.

## Code availability

Code is available upon request from the first authors.
