# Supplementary material for: Machine learning explains response variability of deep brain stimulation on Parkinson’s disease quality of life
Source: NPJ Digit Med. 2024 Oct 2;7:269. doi: 10.1038/s41746-024-01253-y (PMC11445542; doi:10.1038/s41746-024-01253-y)
Supplement: Supplementary file 1 — Supplementary Information [file 41746_2024_1253_MOESM1_ESM.pdf]

Supplementary Information

Supplementary Table 1: Summary Table of Model Parameters and Performances

This table summarizes various model parameters and their corresponding performances. The performance metrics include the Pearson's correlation coefficient (r), mean squared error (MSE), and the p-value of the fitted model. Additionally, the table lists the features sorted by importance, starting from the most important feature (First Feature) to the least important feature (Twelfth Feature).

Supplementary table 2: Summary table of hyperparameter tuning

This table shows the hyperparameter values estimated during the nested leave-one-out optimization method.

Supplementary Table 1

model_name	r	mse	p	First Feat	Second Feat	Third Feat	Fourth Feat	Fifth Feat	Six Feat
Original model (along trajectory)	0.49	0.0933	4.596e-05	Beta_21-35Hz_L	PDQ39_score_pre	LEDD_ratio	Age	TimeSinceSurgery	z_R
Final position (all contacts)	0.36	0.1096	4.282e-03	PDQ39_score_pre	Beta_21-35Hz_L	LEDD_ratio	Age	TimeSinceSurgery	Alpha_8-12Hz_L
Active contacts	0.35	0.1133	4.923e-03	PDQ39_score_pre	Beta_21-35Hz_L	Theta_3-7Hz_L	LEDD_ratio	Age	TimeSinceSurgery
Along trajectory (average of both sides)	0.4	0.1062	1.279e-03	PDQ39_score_pre	Beta_21-35Hz_L	LEDD_ratio	Age	z	TimeSinceSurgery
Affected hemisphere	0.34	0.1111	6.546e-03	PDQ39_score_pre	LEDD_ratio	Beta_13-20Hz_noDom	TimeSinceSurgery	Age	Beta_21-35Hz_Dom
Absolute scores	0.44	0.8243	2.968e-04	PDQ39_score_pre	TimeSinceSurgery	z_L	LEDD_ratio	Beta_21-35Hz_L	Age
Distance and amplitude	0.44	0.0996	2.609e-04	PDQ39_score_pre	Beta_21-35Hz_L	LEDD_ratio	Age	TimeSinceSurgery	L_dist
Model with 12 features	0.55	0.086	3.50E-06	PDQ39_score_pre	Beta_21-35Hz_L	LEDD_ratio	Age	TimeSinceSurgery	z_R

model_name	r	mse	p	Seventh Feat	Eight Feat	Nineth Feat	Tenth Feat	Eleventh Feat	Twelveth Feat
Original model (along trajectory)	0.49	0.0933	4.596e-05	z_L	Theta_3-7Hz_L	beta_low__mean_L	Alpha_8-12Hz_R	Beta_13-20Hz_R	Alpha_8-12Hz_L
Final position (all contacts)	0.36	0.1096	4.282e-03	z_R	Theta_3-7Hz_L	z_L	Alpha_8-12Hz_R	Beta_13-20Hz_L	x_R
Active contacts	0.35	0.1133	4.923e-03	Alpha_8-12Hz_L	z_R	Theta_3-7Hz_R	Beta_13-20Hz_L	y_L	z_L
Along trajectory (average of both sides)	0.4	0.1062	1.279e-03	x	Alpha_8-12Hz	Beta_13-20Hz	Theta_3-7Hz	y	Disease_duration
Affected hemisphere	0.34	0.1111	6.546e-03	z_R	Beta_21-35Hz_noDom	Alpha_8-12Hz_noDom	Theta_3-7Hz_noDom	z_L	y_R
Absolute scores	0.44	0.8243	2.968e-04	Beta_13-20Hz_L	z_R	Theta_3-7Hz_L	Beta_13-20Hz_R	Disease_duration	x_R
Distance and amplitude	0.44	0.0996	2.609e-04	Theta_3-7Hz_L	A_R	Beta_13-20Hz_L	R_dist	Alpha_8-12Hz_R	mean_dist
Model with 12 features	0.55	0.086	3.50E-06	z_L	Theta_3-7Hz_L	Beta_13-20Hz_L	Alpha_8-12Hz_R	Alpha_8-12Hz_R	Beta_13-20Hz_R

Supplementary Table 2

Left out PatID	mse	pearson	reg_alpha	colsample_bytree	subsample	gamma	max_depth	min_child_weight	learning_rate	n_estimators	eval_metric
1	0.083655962	0.554067838	0.004933966	0.679824604	0.829281818	0.030751486	2	10.5339829	0.025	189	rmse
2	0.082492625	0.541463674	0	0.988011636	0.842695963	0.058898586	4	9.631485331	0.025	144	rmse
3	0.083688123	0.561626017	0	0.99815832	0.775713483	0.034538093	6	5.82008788	0.025	80	rmse
4	0.087397447	0.518584331	0	0.83604585	0.984320403	0.012201218	7	9.05382708	0.025	117	rmse
5	0.085790709	0.570506522	0	0.975465503	0.851470946	0.019187485	3	8.727021031	0.025	114	rmse
6	0.086497481	0.52995023	0.001552513	0.999343849	0.73735193	0.044041722	9	8.380326118	0.025	123	rmse
7	0.085192149	0.552695462	0.029123268	0.945334392	0.835621498	0.061352881	3	10.82450461	0.025	157	rmse
8	0.079352495	0.559715962	0	0.943295861	0.799545396	0.003374807	9	8.82010562	0.025	136	rmse
9	0.087139581	0.54588015	0	0.948862908	0.834136817	0.045406825	2	9.966001255	0.025	139	rmse
10	0.083633331	0.599571487	0.154464748	0.953946979	0.760942519	3.15057E-05	4	1.008431172	0.025	89	rmse
11	0.089897749	0.562360978	0.13309095	0.941108933	0.795329225	3.15057E-05	9	6.330541509	0.025	112	rmse
12	0.087158642	0.552353997	0	0.936153349	0.809406211	0.084139105	10	8.807928994	0.025	144	rmse
13	0.087340892	0.554670013	0	0.949150845	0.819246873	0.066590862	2	11.92722829	0.025	157	rmse
14	0.090958484	0.545544533	0.008888062	0.854139331	0.806372965	0.043650415	2	9.328561735	0.025	197	rmse
15	0.090855397	0.519119048	0.00170341	0.985425405	0.933543384	0.050355963	6	8.062373355	0.025	72	rmse
16	0.086207214	0.507699585	0.043416089	0.998948586	0.950857119	0.050355963	2	10.18350048	0.025	182	rmse
17	0.087803141	0.535191777	0	0.983163473	0.815548179	0.097872321	6	9.463184306	0.025	132	rmse
18	0.087231549	0.553178907	0.011227692	0.967074063	0.806917941	0.065415992	4	9.020723662	0.025	157	rmse
19	0.089295943	0.535049102	0	0.949507242	0.802522802	0.060168207	7	10.1561052	0.025	175	rmse
20	0.089416287	0.54179876	0.009086003	0.67405754	0.779250695	0.031487488	7	8.218039776	0.025	192	rmse
21	0.087462721	0.555000698	0.040487545	0.942346123	0.799793924	0.008507838	3	6.604859483	0.025	105	rmse
22	0.089445926	0.531879688	0	0.945347374	0.637664491	0.029051064	3	6.572872119	0.025	132	rmse
23	0.089619935	0.514069039	0	0.986573053	0.819002666	0.054063027	2	10.73827001	0.025	159	rmse
24	0.08696667	0.553941206	0.018691233	0.839928918	0.638184402	0.024711316	8	6.062678327	0.025	133	rmse
25	0.084808661	0.589912042	0.010434746	0.88107113	0.976904456	0.066764232	9	10.86509878	0.025	146	rmse
26	0.090643583	0.56288538	0.000173822	0.815738563	0.676375584	0.005294991	4	6.629170807	0.025	146	rmse
27	0.090261354	0.600818782	0	0.848864103	0.621670281	0.057787805	3	6.042327208	0.025	132	rmse
28	0.090792875	0.552805169	0.043091935	0.709016751	0.623031485	0.071971588	3	6.239122292	0.025	173	rmse
29	0.089735221	0.552655354	0.006190892	0.998110695	0.806792474	0.100845979	6	8.230685948	0.025	107	rmse
30	0.081277235	0.617856042	0.001015864	0.722919351	0.824993107	0.061224198	4	7.378296266	0.025	164	rmse
31	0.090273391	0.540277724	0	0.8	0.8	0.21382844	3	3.363384734	0.025	77	rmse
32	0.091407917	0.512678587	0.012668588	0.890473095	0.924680691	0.059971658	9	6.550241784	0.025	66	rmse
33	0.088083238	0.489521109	0.009178102	0.934758914	0.812831806	0.148181962	5	7.621389947	0.025	67	rmse
34	0.08727662	0.538999424	0.000721983	0.991486825	0.80259252	0.076249967	10	8.209697759	0.025	87	rmse
35	0.069134601	0.64492503	0.000503509	0.647269052	0.869386552	0.058408401	2	10.3568621	0.025	156	rmse
36	0.068549396	0.615645259	0.041346794	0.982229956	0.982042699	0.058408401	2	10.3436796	0.025	148	rmse
37	0.077452317	0.597054498	0	0.685025241	0.801627778	0.099051013	2	9.146488023	0.025	96	rmse
38	0.077653785	0.597978588	0	0.640512799	0.662059297	0.019141009	5	7.677662151	0.025	339	rmse
39	0.077242551	0.581629229	0.019172326	0.708615492	0.810505532	0.019141009	7	10.97027738	0.025	192	rmse
40	0.07089664	0.589789298	0.028376254	0.60245219	0.985799045	0.035091394	2	11.04974962	0.025	1000	rmse
41	0.076054539	0.581635544	0	0.537894219	0.728209903	0.093377844	4	8.548094784	0.025	306	rmse
42	0.080351747	0.597767083	0.005710628	0.806477712	0.798947217	0.088032218	6	8.850211124	0.025	119	rmse
43	0.086687268	0.542028793	0.057805302	0.716572918	0.855194779	0.04033737	5	7.590103582	0.025	164	rmse
44	0.086029175	0.563500833	0	0.687090545	0.779131525	0.000833421	3	8.597221501	0.025	243	rmse
45	0.092029402	0.595158572	0	0.713847403	0.692281115	0.10845246	3	5.784613516	0.025	178	rmse
46	0.095336791	0.434700206	0	0.95216323	0.929545194	0.028899571	6	7.538354319	0.025	120	rmse
47	0.083230869	0.606760041	0	0.912124768	0.783696547	0.105040625	8	7.629714654	0.025	203	rmse
48	0.085015288	0.553207332	0.043011311	0.996887297	0.924773762	0.049370705	7	7.419270533	0.025	60	rmse
49	0.088808418	0.556637351	0.020418632	0.868853512	0.781096774	0.05956065	3	6.876906786	0.025	50	rmse
50	0.089674674	0.557588295	0.001019314	0.909851843	0.784397315	0.111128788	3	7.654049995	0.025	49	rmse
51	0.086013515	0.579708926	0.08233547	0.996465844	0.923911011	0.074780096	4	8.355050272	0.025	60	rmse
52	0.094187888	0.498860833	0	0.998911484	0.855738285	0.123174049	3	7.231057356	0.025	58	rmse
53	0.08784184	0.573030281	0.00329485	0.849847779	0.783252717	0.115961387	8	7.908750585	0.025	60	rmse
54	0.090112274	0.505295007	0	0.980861104	0.836984	0.015689114	6	8.042774426	0.025	93	rmse
55	0.090711531	0.552038101	0.00579627	0.987094551	0.816949415	0.009037672	3	7.455226185	0.025	112	rmse
56	0.081169693	0.607698063	0.005545385	0.989578492	0.853657348	0.000974912	6	7.476361529	0.025	78	rmse
57	0.085897341	0.568713901	0.039383177	0.948908132	0.854894155	0.067336508	6	8.244609355	0.025	101	rmse
58	0.087163679	0.570171114	0	0.939132524	0.839400317	0.097223974	5	8.203536894	0.025	101	rmse
59	0.08425872	0.561618888	0	0.935359234	0.840716947	0.053538633	8	7.683573252	0.025	78	rmse
60	0.08761986	0.535719378	0	0.965277676	0.900871702	0.063790869	6	8.793606006	0.025	128	rmse
61	0.088356739	0.536283376	0	0.849319208	0.854602001	0.003407958	3	5.969802848	0.025	79	rmse
62	0.089856422	0.586392406	0.033334465	0.8	0.8	0.044826017	6	3.097155255	0.025	57	rmse
63	0.086913005	0.544805891	0	0.845321706	0.98645482	0.031179557	9	6.715574109	0.025	31	rmse
**mean**	0.085925564	0.556994774	0.014717383	0.877263484	0.819852727	0.055769875	5.031746	8.040990373	0.025	142.2380952	
**Std**	0.005440306	0.034893442	0.029308499	0.121731771	0.086720242	0.040260104	2.4294447	1.958128486	2.79785E-17	124.1163235	
